# P2Y12 Inhibitors Refill Gap Predicts Death in Medicare Beneficiaries on Chronic Dialysis

**DOI:** 10.1016/j.ekir.2024.04.053

**Published:** 2024-05-07

**Authors:** Rafia S. Rasu, Milind A. Phadnis, Christy Xavier, Junqiang Dai, Suzanne L. Hunt, Nishank Jain

**Affiliations:** 1 Department of Pharmacotherapy, College of Pharmacy, University of North Texas Health Sciences, Fort Worth, Texas, USA; 2 Department of Biostatistics and Data Science, University of Kansas School of Medicine, Kansas City, Kansas, USA; 3 Department of Internal Medicine, University of Arkansas for Medical Sciences, Little Rock, Arkansas, USA; 4 Central Arkansas Veterans Affairs Medical Center, Little Rock, Arkansas, USA

**Keywords:** dialysis, disparities, medicare, refill gap

## Abstract

**Introduction:**

Oral P2Y_12_ inhibitors (P2Y12-I) are commonly used antiplatelet drugs in patients with end-stage kidney disease (ESKD) on chronic dialysis. Although gaps in prescription refills are quite common in patients with ESKD, it remains unclear whether P2Y12-I prescription refill patterns are associated with adverse clinical outcomes.

**Methods:**

We used the United States Renal Data System (USRDS) registry for patients with ESKD to capture new P2Y12-I prescriptions from 2011 to 2015. The primary exposure was prescription refill patterns and the primary outcome was all-cause death.

**Results:**

Among the 31,243 patients with new P2Y12-I prescription, median age was 64 years; 54% were male; and 39% were Caucasian, 37% African American, and 18% Hispanic. We observed 3 P2Y12-I refill patterns as follows: continuous users (45.1%), noncontinuous users (3.6%), and users with ≥30 days refill gap (51.4%). Prescription refill pattern with ≥30 days refill gap (vs. continuous use) was associated with all-cause death (adjusted hazard ratio [HR]: 1.18; 95% confidence interval [CI]: 1.13–1.23). Age and race were the most important risk factors associated with prescription refill pattern. African Americans (vs. Caucasians) were more likely to demonstrate ≥30 days refill gap, (adjusted odds ratio [OR]: 1.43; 95% CI: 1.36–1.51). In addition, younger patients (vs. older) were more likely to demonstrate ≥30 day refill gap (adjusted OR/decade: 0.9; 95% CI: 0.89–0.92).

**Conclusion:**

Nonadherence to P2Y12-I prescriptions is quite common, and disproportionately affects minorities. Younger individuals with ESKD are independently associated with a higher risk of death. The odds of having a refill gap are decreasing for older patients who are more compliant than younger patients. Future studies should investigate whether phenotyping subgroups of patients with ESKD based on prescription refill patterns can help in improving adverse clinical outcomes.

## Graphical abstract

**Figure:**
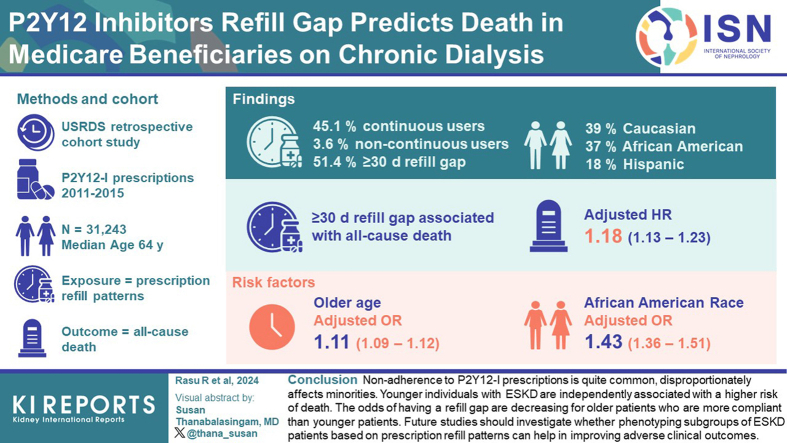

Chronic kidney disease is a progressive renal disease with high clinical, humanistic, and economic burden. In 2019, 37 million Americans had chronic kidney disease, and 786,000 Americans had ESKD receiving maintenance dialysis. Medicare beneficiaries with kidney diseases accounted for 25% of the total Medicare expenditures in 2017.^1^^,^^2^ Most of these expenses are related to treating cardiovascular (CV) events in these patients. Oral P2Y12-Is are also commonly prescribed to treat CV events in this patient population; this prescription class is amongst the top 10 most prescribed drugs for patients with ESKD. Despite these prescriptions, CV-death accounts for approximately half of all-deaths in patients with ESKD. Of the many reasons for excessive CV risk in this patient population despite guideline-based treatment, nonadherence to prescriptions appears to be a common problem among patients with ESKD. Nearly 20% of patients exhibit nonadherence at 30 days of receiving a new prescription for P2Y12-I; a number that increases to 27.5% at 6 months.^2^ Despite nonadherence to P2Y12-I prescriptions being a common problem, its association with death remains unclear in this patient population. Moreover, risk factors associated with nonadherence remains poorly understood for these patients.

It is crucial to identify risk factors for nonadherence to P2Y12-I prescriptions, and its association with death among patients with ESKD. Affordable care organizations and payers are increasingly relying on tracking patients electronically to identify “at-risk” individuals by e-phenotyping them,^3^ so as to offer value-based care, reduce health care spending and reduce adverse clinical outcomes.^4^^,^^5^ Patients with ESKD often fall in the “high-risk” category, that is, high-utilizer of health care services that negatively affects value-based reimbursement from Medicare and other payers.^6^, ^7^, ^8^ Identifying risk factors for nonadherence to a commonly prescribed drug such as P2Y12-I prescriptions, and its association with death is crucial to help affordable care organizations in designing and implementing intervention strategies for mitigating nonadherence in a “high-risk” patient population-ESKD patients. The objective of this study was to investigate whether nonadherence, as defined by refill gaps in P2Y12-I prescriptions, are associated with all-cause death among patients with ESKD. We also wanted to identify risk factors for nonadherence, and identify subgroups of patients with ESKD who might be more vulnerable to nonadherence. To achieve our objectives, we used readily available data from the USRDS registry of individuals with ESKD.

## Methods

### Data Source

We used data from USRDS, a national registry of patients with ESKD that includes demographic and comorbidity conditions documented upon initiation of dialysis; dialysis treatment type over time; date and cause(s) of death; and patient-level Medicare institutional (Part A), physician-supplier (Part B), and prescription drug (Part D) claims. Patient-level demographic data, clinical data, dialysis modality, and first service date of dialysis is generated upon initiation of dialysis, when nephrologists are required to submit a Medical Evidence Form (CMS-2728) to regional ESKD networks. Each ESKD network then forwards the information to the USRDS Coordinating Center and continues to provide regular patient updates. Medicare Part A claims include dates of admissions to hospitals, primary diagnosis, procedures performed during hospitalization, and contributing or concurrent diagnoses (based on International Classification of Diseases 9 [ICD-9-CM] codes and diagnosis-related grouping) for each admission. Medicare Part B claims include details of outpatient services (e.g., dates of service, ICD-9-CM diagnosis, and procedure codes). Medicare Part D claims include details of prescription drug use (e.g., P2Y12-I type, strength, and days’ supply).

### Study Design and Cohort

Institutional review board approvals were obtained from the University of Arkansas for Medical Sciences, Little Rock, Arkansas, and University of Kansas Medical Center, Kansas City, Kansas (primary site for data collection, processing, and analysis). Subsequently, a data-use agreement was signed and approved by the USRDS Program Director (DUA 2016-50). From administrative claim files dated January 1, 2011, through December 31, 2015, we created a retrospective national cohort of patients with prevalent ESKD. Our observation period started July 20, 2011, when ticagrelor became available in the market; it ended on September 30, 2015, just before the transition from ICD-9 to ICD-10 codes. After applying exclusions, we identified patients in the dataset who were continuously eligible for Medicare Parts A, B, and D. From the start of the observation period, we identified new prescriptions for P2Y12-I; the date of first prescription was called the index date. Two steps were used to identify new prescriptions. First, prescriptions for P2Y12-I were identified from nonproprietary drug names in Medicare Part D claims. Second, any prescription that appeared after a 6-month period with no P2Y12-I exposure was assumed to be a new prescription. Because pharmacologic effects of P2Y12-Is wash out relatively quickly, 6 months without exposure seemed suitable for defining new prescriptions.

The study cohort included patients undergoing hemodialysis or peritoneal dialysis (Supplementary Figure S1 and STROBE Checklist). Those who received transplants but returned to dialysis because of failed allografts before index dates were considered for inclusion. We included any patient who was aged at least 18 years; was receiving maintenance dialysis; had survived at least 6 months from the first USRDS-recorded service; was continuously eligible for Medicare Parts A, B, and D 6 months before the index dates; and received new prescriptions for P2Y12-Is. A patient was excluded if aged younger than 18 years; date of first USRDS-recorded service was missing; dialysis started after the study end date; eligibility for Medicare Parts A, B, and/or D was noncontinuous; chronic dialysis treatments were not received; P2Y12-I was not prescribed; or P2Y12-I prescription was not new. When 2 different P2Y12-Is were prescribed on index dates (*n* = 3), the P2Y12-I observed in the subsequent prescription was considered the index drug.

### Outcome Variable

The primary outcome was time-to-all-cause death. We calculated the time from an index date to the date of death. All-cause death was ascertained from the USRDS registry; it is a reliable source to capture deaths for patients with ESKD.^2^

### Exposure Variable

Part D prescription claims for P2Y12-Is, first fill date, and days’ supply were flagged. The index date was defined as the date of the new P2Y12-I prescription. The earliest possible index date was July 20, 2011, when ticagrelor became available. All cohort members were followed-up with from the index date (start of P2Y12) to their time-to-death (or end of study). There was no separate “adherence evaluation period.” The categorization of the cohort into the 3 different refill gap patterns was not done in a separate “evaluation time period.” The only qualifying criteria for the subjects in our study was that they have survived at least 180 days. Measure of adherence was hospital-adjusted proportion of days covered (HA-PDC) that was calculated with prescription refills and days’ supply and, extending prescription run-out dates for overlaps in refills and hospitalizations. HA-PDC makes the cacophony of adherence measures clearer and more accurate. Adjusting proportion of days covered for refill overlaps and hospital length of stay affected 41% and 52.7% of the cohort, respectively. Due to the high rate of hospitalization among this cohort (78%) with median length of stay of 12 days (interquartile range: 2–34), our HA-PDC is a more appropriate measure to capture P2Y12-I adherence in patients with ESRD.^2^

### Covariates

Demographics data was collected from CMS-2728 (recorded at first USRDS service). Information related to dialysis treatment, including modality (hemodialysis vs. peritoneal dialysis), vintage (time between dialysis initiation and index date), and underlying cause of ESKD, was collected. Comorbidities were collected from CMS-2728 and combined with codes appearing on 2 different days in outpatient claims data or once in hospital claims data 6 months before the index date. Details of ICD-9, diagnosis-related group, current procedural terminology, and health care common procedure coding system codes that were used to identify comorbidities are provided. Modified Liu comorbidity index, a validated measure of comorbidity burden in the ESKD patient population, was calculated using algorithm previously published.^1^^,^^2^

### Censoring

The cohort was followed-up with until death, kidney transplantation, switching between P2Y12-Is, initiation of oral anticoagulants, lost to USRDS follow-up, or discontinuation of Medicare Part D. If none of these events occurred, follow-up continued until September 30, 2015. Thus, when a member switched between 3 P2Y12-Is medications, marked as censored. Detailed switching of these 3 agents are shown in the STROBE checklist.

### Statistical Analyses

Summary statistics were reported using counts and frequencies for categorical variables, and using means and SDs for continuous variables. For skewed variables, median and interquartile range was used to summarize data and represented graphically using grouped box plots. A logistic regression was performed to assess the association between adherence patterns (gap users vs. nongap users) and various risk factors. The primary outcome of death was analyzed using a Cox proportional hazards model. First, unadjusted HRs along with corresponding 95% CIs were calculated in a univariable model comparing across the 3 HA-PDC categories (≥30 day gap, continuous users, infrequent users). Next, adjusted HRs with corresponding 95% CIs were calculated in a sequential way, adjusting for demographic variables, dialysis-related risk factors, and for all other comorbidities. The *P* values for these HRs were adjusted for multiplicity using the Bonferroni correction method. Using our final model, we identified the top risk factors associated with nonadherence. An interaction between risk factor and adherence was added to our Cox model to assess the impact of the risk factor in modifying the effect of adherence over all-cause death. For all Cox models, the validity of the proportional hazards assumption was assessed using Schoenfeld residuals for continuous covariates, and using “observed versus expected” plots for categorical variables. All analysis was conducted using the SAS version 9.4 software (SAS Institute Inc., Cary, NC; https://www.sas.com/en_us/home.html). Type I error was set at the 5% level of significance.

## Results

There were 160,793 patients who received P2Y12-I prescriptions during the observation period. After application of exclusion criteria and identifying new users, 31,243 new P2Y12-I users were included in the cohort. Supplementary Figure S1 Consort diagram and STROBE checklist show the derivation of the study cohort. Median (interquartile range) age of the cohort was 64 (55–72) years. There were 53.5% men, 38.7% Caucasians, 37% African Americans, and 18% Hispanics. Almost three-quarters of cohort (75.09%) belonged to low-income subsidy category, one of the strongest and well-established predictor of socioeconomic status for Medicare beneficiaries in the US. Of the patients 93.5% received hemodialysis and the remaining 6.5% received peritoneal dialysis treatments. The median time on dialysis was 3.8 (1.9–6.6) years. The median number of concomitant medications at index dates was 7 (5–10). Of the cohort, 8.2% were receiving anticoagulants at index dates. In addition, 27.5% of the cohort had claims available for percutaneous coronary interventions within 6 months prior to index dates. Median follow-up was 547 (interquartile range: 335–863) days with a total of 53,794.5 person-years.

### Pattern of P2Y12-I Prescriptions

**Figure 1:**
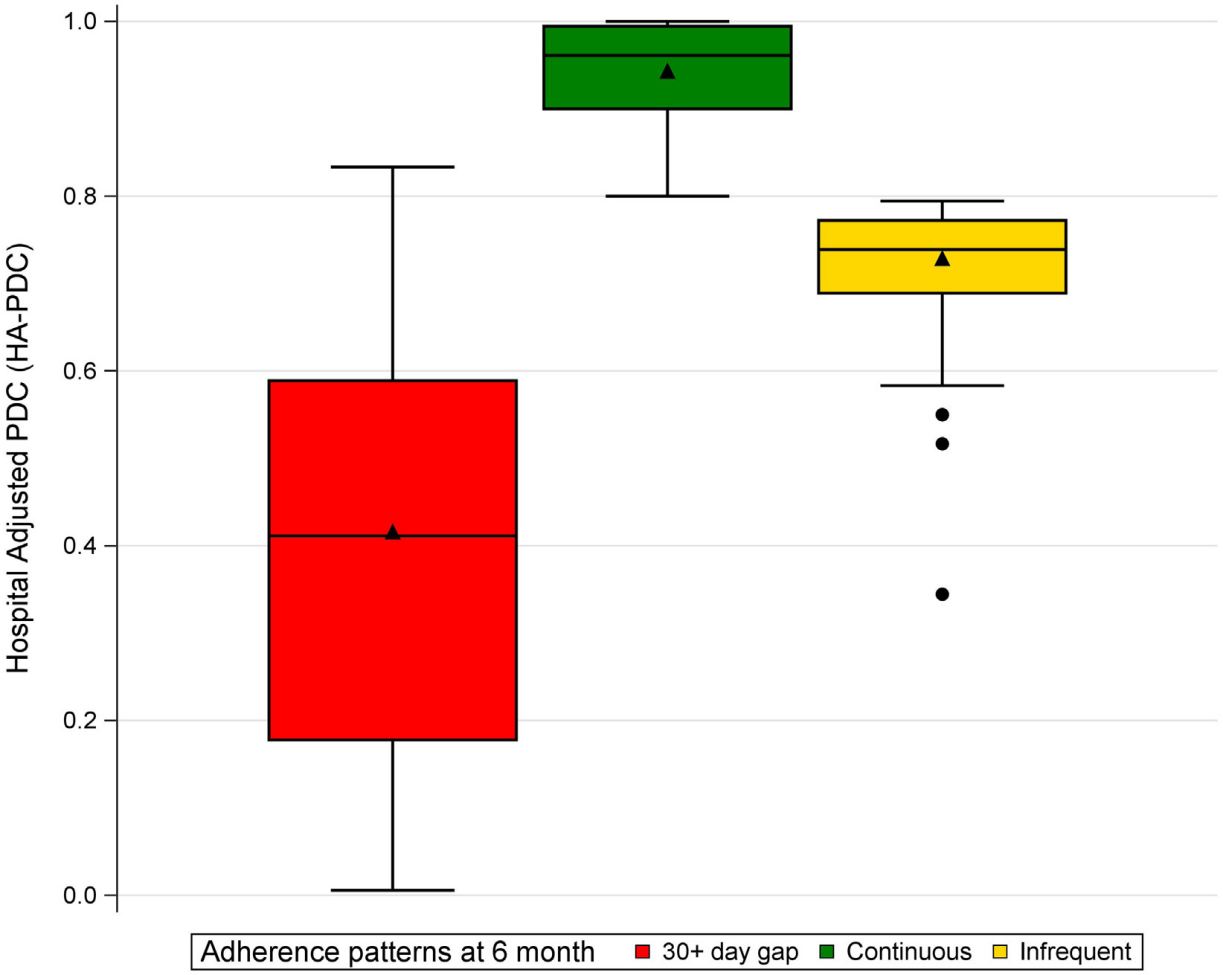
Patterns of P2Y12-I prescriptions adherence. PDC, proportion of days covered.

Median HA-PDC was 0.74 (0.39–0.96) for the entire cohort. Three categories of adherence were identified in the cohort at 6 months (Figure 1) as follows: (i) continuous users without any refill gaps of ≥30 consecutive days and HA-PDC of ≥0.8, (ii) infrequent users without any refill gaps of ≥30 consecutive days and HA-PDC of <0.8, and (iii) users with ≥30 days refill gap regardless of HA-PDC value. There were 45.1% users (*n* = 14,084) with continuous-use refill pattern (median HA-PDC, 0.96 [0.90–0.99]); and 51% (*n* = 16,044) were users with ≥30 days refill gap (median HA-PDC, 0.41 [0.18–0.59]) (Table 1). The remaining 3.6% (*n* = 1115) were infrequent users (median HA-PDC, 0.74 [0.69–0.77]) (Table 1). Most of the 79.55 % of infrequent users availed low-income subsidy, 73.77% in continuous category, and 75.93% in ≥30 days refill gap category.

**Table 1:** Characteristics of the study population

Characteristics	All *N* = 31,243	30+ days gap *n* = 16,044	Continuous *n* = 14,084	Infrequent *n* = 1115	30+ days gap vs. continuous Std. diff (%)	Infrequent vs. continuous Std. diff (%)
Demographics						
Age, yr^a^	64.0 (55.0–72.0)	63.0 (54.0–71.0)	65.0 (56.0–73.0)	62.0 (54.0–70.0)	−15.6	−20.3
Male gender	16,719 (53.5%)	8525 (53.1%)	7602 (54.0%)	592 (53.1%)	−1.7	−1.8
Ethnicity, Hispanic/Latino	6042 (19.3%)	3169 (19.8%)	2641 (18.8%)	232 (20.8%)	2.5	5.0
Race						
African American	11,570 (37.0%)	6538 (40.8%)	4558 (32.4%)	474 (42.5%)	24.9	30.1
Caucasian	12,100 (38.7%)	5620 (35.0%)	6117 (43.4%)	363 (32.6%)		
Hispanics	5778 (18.5%)	3027 (18.9%)	2527 (17.9%)	224 (20.1%)		
Other races	1731 (5.5%)	827 (5.2%)	854 (6.1%)	50 (4.5%)		
History of smoking tobacco	2144 (6.9%)	1150 (7.2%)	901 (6.4%)	93 (8.3%)	3.1	7.6
Low-income subsidy	23,459 (75.1%)	12,182 (75.9%)	10,390 (73.8%)	887 (79.6%)	5.0	13.7
Dialysis-related factors						
Dialysis modality						
Hemodialysis	29,213 (93.5%)	15,081 (94.0%)	13,075 (92.8%)	1057 (94.8%)	4.7	8.2
Peritoneal dialysis	2030 (6.5%)	963 (6.0%)	1009 (7.2%)	58 (5.2%)		
Yrs on dialysis	3.8 (1.9–6.6)	3.9 (2.0–6.8)	3.6 (1.8–6.4)	3.8 (1.9–6.4)	4.9	0.3
Anticoagulant use on index date	2553 (8.2%)	1382 (8.6%)	1101 (7.8%)	70 (6.3%)	2.9	−6.0
Modified Liu index	7.0 (4.0–9.0)	7.0 (4.0–9.0)	6.0 (4.0–9.0)	7.0 (4.0–10.0)	−0.1	4.2
Comorbidities						
Hypertension	28,060 (89.8%)	14,370 (89.6%)	12,683 (90.1%)	1007 (90.3%)	−1.5	2.2
Diabetes mellitus	24,447 (78.2%)	12,494 (77.9%)	11,069 (78.6%)	884 (79.3%)	−1.5	1.9
Cancer	2623 (8.4%)	1304 (8.1%)	1240 (8.8%)	79 (7.1%)	−2.4	−6.4
Liver disease	2372 (7.6%)	1282 (8.0%)	992 (7.0%)	98 (8.8%)	3.6	6.5
GI bleeding	2256 (7.2%)	1206 (7.5%)	964 (6.8%)	86 (7.7%)	2.6	3.3
COPD	9247 (29.6%)	4768 (29.7%)	4141 (29.4%)	338 (30.3%)	0.7	2.0
AMI	6661 (21.3%)	3024 (18.8%)	3385 (24.0%)	252 (22.6%)	−12.7	−3.4
STEMI	3251 (10.4%)	1435 (8.9%)	1678 (11.9%)	138 (12.4%)	−9.7	1.4
NSTEMI	5513 (17.6%)	2515 (15.7%)	2782 (19.8%)	216 (19.4%)	−10.7	−1.0
Cardiogenic shock or cardiac arrest	2299 (7.4%)	1158 (7.2%)	1038 (7.4%)	103 (9.2%)	−0.6	6.8
CABG	827 (2.6%)	446 (2.8%)	350 (2.5%)	31 (2.8%)	1.8	1.8
PCI performed	8591 (27.5%)	3241 (20.2%)	5014 (35.6%)	336 (30.1%)	−34.9	−11.7
Coronary stent(s) deployed	8333 (26.7%)	3094 (19.3%)	4913 (34.9%)	326 (29.2%)	−35.7	−12.1
Multiple coronary stents deployed	4154 (13.3%)	1560 (9.7%)	2405 (17.1%)	189 (17.0%)	−21.7	−0.3
Drug-eluting stent deployed	4515 (14.5%)	1447 (9.0%)	2876 (20.4%)	192 (17.2%)	−32.6	−8.2
Bare-metal stent deployed	7943 (25.4%)	2982 (18.6%)	4656 (33.1%)	305 (27.4%)	−33.5	−12.4
Atrial fibrillation	5578 (17.9%)	2829 (17.6%)	2560 (18.2%)	189 (17.0%)	−1.4	−3.2
Congestive heart failure	18,674 (59.8%)	9533 (59.4%)	8448 (60.0%)	693 (62.2%)	−1.2	4.5
Abnormal stress test	909 (2.9%)	388 (2.4%)	492 (3.5%)	29 (2.6%)	−6.4	−5.2
Peripheral vascular disease	11,167 (35.7%)	6972 (43.5%)	5421 (38.5%)	470 (42.2%)	10.1	7.5
Amputation	1330 (4.3%)	820 (5.1%)	453 (3.2%)	57 (5.1%)	9.5	9.5
Ischemic stroke	3719 (11.9%)	1817 (11.3%)	1745 (12.4%)	157 (14.1%)	−3.2	4.9
Intracranial hemorrhage	285 (0.9%)	159 (1.0%)	116 (0.8%)	10 (0.9%)	1.8	0.8
Number of comorbidities	5.0 (3.0–7.0)	4.0 (3.0–7.0)	5.0 (3.0–8.0)	5.0 (3.0–8.0)	−25.2	−3.4
Concomitant medicines						
Number of concomitant medicines index date	7.0 (5.0–10.0)	7.0 (5.0–9.0)	8.0 (6.0–10.0)	7.0 (5.0–9.0)	−25.3	−26.5
Antihypertensive medicine	23,254 (74.4%)	11,425 (71.2%)	11,039 (78.4%)	790 (70.9%)	−13.7	−10.3
Statins	15,909 (50.9%)	7264 (45.3%)	8090 (57.4%)	555 (49.8%)	−16.6	−17.4
Proton pump inhibitor	9557 (30.6%)	4442 (27.7%)	4789 (34.0%)	326 (29.2%)	−24.5	−15.4

AMI, acute myocardial infarction; CABG, coronary artery bypass grafting; COPD, chronic obstructive pulmonary disease; GI, gastrointestinal; NSTEMI, non-ST-elevated myocardial infarction; PCI, percutaneous coronary interventions; STEMI, ST-elevated myocardial infarction; Std. diff., standardized difference.

All Eligibility was tabulated for 6 months prior to the index date.

a Ages were calculated on index date; and reported as median (range).

### Risk Factors Associated With Prescription Refill Patterns

**Figure 2:**
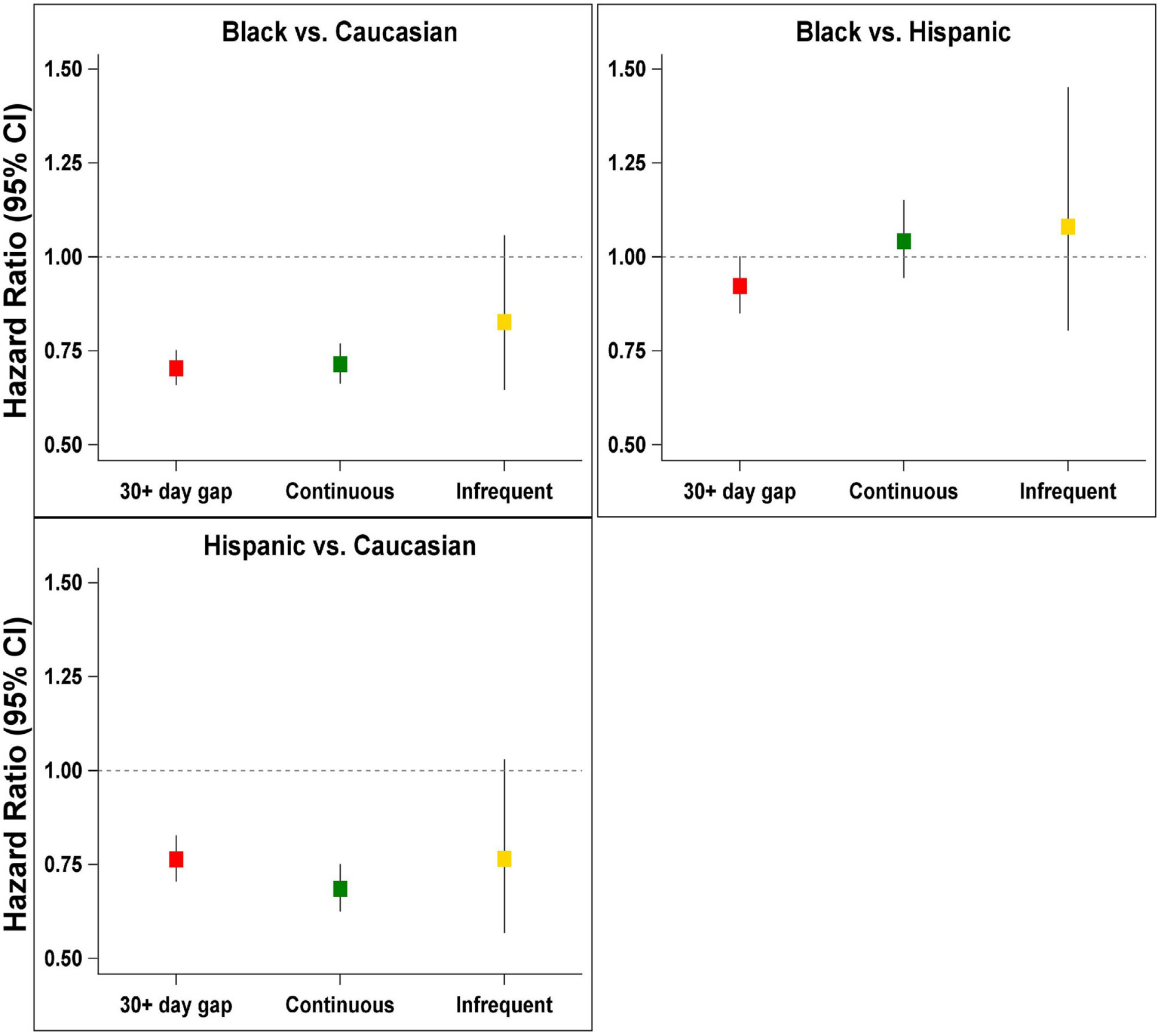
Comparison of races with refill patterns. CI, confidence interval.

Race and age were the most significant risk factors associated with prescription refill pattern in patients with ESKD (Figure 2). Specifically, African Americans (vs. Caucasians) were more likely to be users with ≥30 days refill gap (adjusted OR: 1.43, 95% CI: 1.36–1.51, multiplicity adjusted *P* < 0.01). No other racial pairwise comparisons were statistically significant. Older patients were more likely to be continuous users (adjusted OR for every 10-year increase in age: 1.10, 95% CI: 1.08–1.12, *P* value < 0.01). Age was the second-most important risk factor (Chi-square: 102.25, *P* value < 0.01) associated with the ≥30 day gap. In addition, younger patients (vs. older) are more likely to demonstrate ≥30 day refill gap (adjusted OR/decade is 0.90; 95% CI: 0.89–0.92). The odds of becoming a gap user are decreasing for older patients who are more compliant than younger patients. Likewise, users with ≥30 days refill gap were more likely to have a history of peripheral vascular disease (PVD) (adjusted OR: 1.27, 95% CI: 1.21–1.33, *P* < 0.01); and were receiving dialysis treatment for a longer duration (adjusted OR/ unit increase in logarithm of years on dialysis: 1.03, 95% CI: 1.01–1.06, *P* = 0.01).

### All-Cause Death

There were 9307 (29.8%) deaths during the observation period; 4932 (30.7%) deaths among 16,044 users with ≥30 days refill gap, 4035 (28.7%) deaths among 14,084 continuous users, and 340 (30.5%) among 1115 infrequent users (Table 2).

**Table 2:** Number of events depicting association of P2Y12 inhibitors prescription refill patterns with death

Outcomes of interest	All *N* = 31,243	30+ days gap *n* = 16,044	Continuous *n* = 14,084	Infrequent *n* = 1115	*P* value
Primary outcomes					
All-cause death, *n* (%)	9307 (29.8)	4932 (30.7)	4035 (28.7)	340 (30.5)	0.0003

### Association of User Patterns With All-Cause Death

Compared to the continuous users, Cox proportional hazards model suggested that there was higher risk of all-cause death among the users with ≥30 days refill gap (unadjusted HR: 1.10; 95% CI: 1.05–1.14). This association persisted even after adjusting for age, years on dialysis, and burden of comorbidities (adjusted HR: 1.18; 95% CI: 1.13–1.23) (Table 3). Unadjusted analysis did not reveal a significant association with all-cause death when comparing infrequent and continuous users (Table 3). Model adjustments for demographics include age, gender, and race; and socioeconomic status includes low-income subsidy. In addition, for this study, we adjusted our model for dialysis-related factors, including dialysis modality and dialysis vintage; and comorbidities including dialysis-related factors, year of index date, history of chronic obstructive pulmonary disease, history of atrial fibrillation, history of ischemic stroke, history of PVD, and modified Liu comorbidity index.

**Table 3:** Cox model showing association between all-cause death and user groups with sequentially added risk factors

Pairwise user-group comparison	Unadjusted		Adjusted					
			Plus, demographics		Plus, dialysis-related factors		Plus, comorbidities	
	HR (95% CI)	*P* value	HR (95% CI)	*P* value	HR (95% CI)	*P* value	HR (95% CI)	*P* value
30+ day gap vs. continuous users	1.095 (1.050–1.142)	<0.0001	1.183 (1.135–1.234)	<0.0001	1.182 (1.134–1.233)	<0.0001	1.180 (1.131–1.231)	<0.0001
Infrequent vs. continuous users	1.042 (0.933–1.164)	0.9999	1.181 (1.056–1.319)	0.0102	1.189 (1.064–1.329)	0.0069	1.151 (1.030–1.287)	0.0405
30+ day gap vs. infrequent users	1.050 (0.941–1.172)	0.9999	1.011 (0.905–1.128)	0.9999	1.000 (0.896–1.117)	0.9999	1.031 (0.923–1.152)	0.9999

CI, confidence interval; HR, hazard ratio; vs., versus.

The following covariates were included in the sequential models:.

Plus, demographics includes age, gender, race and low-income subsidy.

Plus, dialysis-related factors includes demographics + dialysis modality + log (dialysis vintage) in days.

Plus, comorbidities includes dialysis related factors + year of index date + history of COPD + history of atrial fibrillation + history of ischemic stroke + history of peripheral vascular disease + modified Liu comorbidity index.

### Assessing Potential Interaction Between Race and Adherence Pattern for All-Cause Death

We did not find a statistically significant interaction by adding a “race pattern” interaction in our Cox model (*P* for interaction = 0.38). That is, the association between race and death did not change depending on the type of adherence pattern as shown in Figure 2.

## Discussion

Although treatment guidelines encourage at least 6 months of P2Y12-I use after prescribing for a clinical indication, nonadherence is common and widely prevalent within 6 months of new P2Y12-I prescriptions in patients with ESKD. Moreover, younger patients and African Americans were more likely to exhibit ≥30 days refill gap in P2Y12-I use even after adjusting for their socioeconomic status. Moreover, nonadherence to P2Y12-I worsens the risk for death in patients with ESKD even after adjusting for multiple risk factors (>15 in the model). Based on our findings, race and age are important risk factors associated with nonadherence to P2Y12-I therapy in patients with ESKD. Our findings identify key determinants of death in patients with ESKD with new P2Y12-I use because recurrent CV events from stent thrombosis and in-stent restenosis are leading causes of death in this patient population. However, our findings also generate an opportunity to identify high risk subgroups; for example, patients with ≥30 days refill gap, where identifying a vulnerable population at-risk for nonadherence provides opportunity to e-phenotype them such that future strategies could focus on implementing mitigating strategies for nonadherence with the hope of improving death among patients with ESKD.

We found that nonadherence to P2Y12-I prescriptions is common and independently associated with all-cause death. Nonadherence to P2Y12-I prescriptions is not a new problem; this is especially high for new prescriptions in the general population and, among individuals with ischemic heart disease.^9^ In the general population, there was a notable increase in the proportion of individuals from 6.4% to 19.1% who did not fill any P2Y12-I prescriptions beyond 30 days of receiving new prescription during hospitalization.^10^ In a Canadian study observing P2Y12-I adherence trajectories over time among patients with acute coronary syndrome who had a percutaneous coronary intervention and a drug-eluting stent, key contributors to major adverse CV events were early consistent nonadherence, delayed initiation of P2Y12-I, and rapid decline of P2Y12-I use after percutaneous coronary intervention.^11^, ^12^, ^13^, ^14^ In our study, we extend the findings observed in the CV literature and report that nearly half of the patients with ESKD with new P2Y12-I use demonstrate nonadherence at 6 months and it is an independent predictor of death.^1^^,^^15^

In addition to widespread prevalence of nonadherence to P2Y12-I prescriptions, we also found certain demographics and comorbidities to be important determinants of nonadherence among patients with ESKD. We found nonadherence in younger patients with ESKD when compared to those who were aged 10 years or older. These patterns are similar to that reported in the CV literature where older age was shown to have better adherence. We also found that ≥30 days refill gap was more likely to occur among African Americans than among Caucasians. This adherence pattern could be due to affordability because minorities were more likely to receive low-income subsidy in our cohort (85.6% of non-Whites received low-income subsidy vs. 58.5% of Whites) and are more likely to have gaps in Medicare Part D prescription coverage that reduces out-of-pocket costs through low-income subsidy. Finally, we found preexisting conditions such as PVD and stroke to be associated with nonadherence to P2Y12-I use among patients with ESKD. Previous investigators found that smoking decreased the odds of P2Y12-I adherence by nearly 55%.^5^^,^^16^ Our study contributes to this growing research by showing that subgroups of patients with ESKD such as those who have physical limitations from PVD, or have cognitive impairment from previous stroke may be more likely to exhibit nonadherence to P2Y12-I prescriptions. Given this information, identifying demographic-based and comorbidity-based adherence pattern provides an opportunity for the affordable care organizations to e-phenotype “high-risk” patients in a given population, and develop mitigation strategies to improve adherence.

In patients who receive coronary stents, nonadherence (>30 days’ gaps in refill) accounts for up to half of the cases of recurrent CV events.^11^ However, previous studies reported the rates of major adverse CV events without reporting the HRs associated with such nonadherence, or they failed to adjust for demographics and comorbidities.^10^ We are extending the findings of previous studies and filling these knowledge gaps. Our risk estimate is also much lower than previously reported in the general population because we adjusted for more covariates (>15) than the previous literature (HR: 1.18 vs. 1.65 for CV event or death).^10^ In summary, the clinical relevance of such a finding is that adherence in high-risk groups such as young minorities portends higher CV risk and should be intervened with electronic tracking of such patients (e-phenotyping them) to improve their health outcomes.

According to a study conducted among Veterans about nonadherence to CV medications in patients on dialysis,^17^ adherence to other concurrent medications was not reported. In other publications in the CV literature,^10^ the nonadherence to a particular medicine of interest did not include adherence to concomitant medicines. For this analysis, we only flagged concurrent medications but did not measure their adherence patterns. On average, cohort members were on 7 different types of medications at index dates, and capturing adherence patterns for each would be beyond the scope of this analysis. Hence, we did not feel the need to study adherence patterns for concomitant medicines in this analysis.

Our study has various limitations. Although we used a rigorous statistical approach, residual confounding could still exist. Second, we used datasets prior to the transition from ICD-9-CM to ICD-10-CM codes. Trends for nonadherence to P2Y12-Is could change with time; and future work could use more recent datasets to confirm our results. Third, P2Y12-Is could be prescribed for various clinical indications, including acute myocardial infarction, stroke, or PVD; this might modify the ill-effects of nonadherence based on clinical indication. We discontinued enrollment when a patient switched between P2Y12-Is medications which may have a potential confounding effect. Another limitation was variable selection. In our analyses, however, 19 of the 26 covariates were very highly statistically significant (*P* values of the order of 0.0001 or 0.001) and the remaining 7 were highly not significant (very high *P* values). Therefore, even if we do not use a variable selection procedure and deploy a model with all covariates, the results remain the same. In addition, our results are limited by potential residual confounding inherent to observational analyses. Despite these limitations, our study is strengthened by use of a large, national, multiethnic cohort of an understudied, high-risk patient population. Because of lack of evidence regarding P2Y12-I use in ESKD, our results become even more relevant. A large pragmatic trial in this patient population can be undertaken with the goal to improve adherence and their lives. Our data and current alarming trends show that current practice is not sufficient to ensure high medication adherence and therapeutic success in patients who have begun taking a new P2Y12-I prescription. Our findings emphasize the need for an early and targeted approach to P2Y12-I therapy in this “high-risk” and vulnerable patient population.

In summary, this study reports that nonadherence to P2Y12-I prescriptions is common among patients with ESKD and is independently associated with death in this patient population. Certain patient characteristics are more likely to be associated with this type of nonadherence among patients with ESKD, including younger age, minorities, and individuals with PVD or stroke. These findings suggest that identifying subgroups of patients with ESKD who are more likely to show nonadherence may be important so that intervention strategies can be developed to mitigate its ill-effects on death in this patient population.

## Disclosure

All the authors declared no competing interests.

## Supplementary Material

Supplementary File (PDF) **Figure S1.** Study population. **STROBE Checklist.**

## Footnotes

Supplementary File (PDF)
**Figure S1.** Study population.
**STROBE Checklist.**
