# Supplementary material for: P2Y12 Inhibitors Refill Gap Predicts Death in Medicare Beneficiaries on Chronic Dialysis
Source: Kidney Int Rep. 2024 May 7;9(7):2125–33. doi: 10.1016/j.ekir.2024.04.053 (PMC11284433; doi:10.1016/j.ekir.2024.04.053)
Supplement: Supplementary File (PDF) — Figure S1. Study population. STROBE Checklist. [file mmc1.pdf]

Figure S1: Study Population

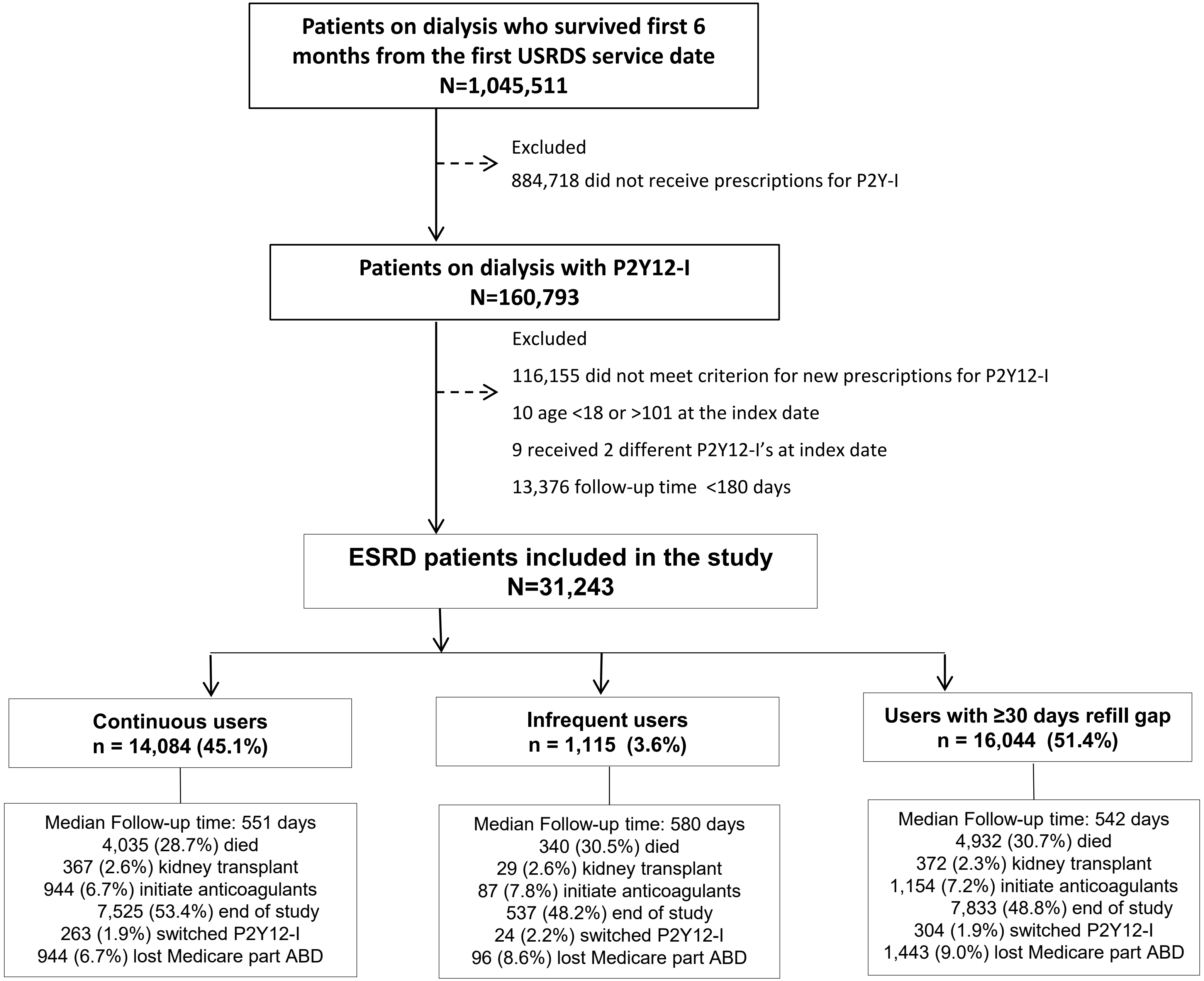

Figure S1:
STROBE checklist
Statement—Checklist of items that should be included in reports of ***cohort studies***

	Item No	Recommendation
**Title and abstract**	1	(*a*) Exposure (gap) predicts outcomes (death)
		(*b*) Provided
**Introduction**		
Background/rationale	2	Provided
Objectives	3	Provided
**Methods**		
Study design	4	Provided
Setting	5	Provided
Participants	6	(*a*) Provided
		(*b*) F Provided
Variables	7	Clearly define- Provided
Data sources/ measurement	8*	Provided
Bias	9	Provided
Study size	10	Provided
Quantitative variables	11	Provided
Statistical methods	12	(*a*) Provided
		(*b*) Provided
		(*c*) Provided
		(*d*) Provided
		(*e*) Provided
**Results**		
Participants	13*	(a) Provided
		(b) Provided
		(c) Consider use of a flow diagram- CONSORT diagram provided
Descriptive data	14*	(a) Provided
		(b) Provided
		(c) Provided
Outcome data	15*	Provided
Main results	16	(a) Provided
		(*b*) Provided
		(*c*) Provided
Other analyses	17	Provided
**Discussion**		
Key results	18	Provided
Limitations	19	Provided
Interpretation	20	Provided
Generalisability	21	Provided
**Other information**		
Funding	22	Provided

*Give information separately for exposed and unexposed groups.

Note: An Explanation and Elaboration article discusses each checklist item and gives methodological background and published examples of transparent reporting. The STROBE checklist is best used in conjunction with this article (freely available on the Web sites of PLoS Medicine at http://www.plosmedicine.org/, Annals of Internal Medicine at

http://www.annals.org/, and Epidemiology at http://www.epidem.com/). Information on the STROBE Initiative is available at http://www.strobe-statement.org.
